# Live *In Vivo* Imaging of *Plasmodium* Invasion of the Mosquito Midgut

**DOI:** 10.1128/mSphere.00692-20

**Published:** 2020-09-02

**Authors:** Nathanie Trisnadi, Carolina Barillas-Mury

**Affiliations:** a Laboratory of Malaria and Vector Research, National Institute of Allergy and Infectious Diseases, National Institutes of Health, Rockville, Maryland, USA; The Hebrew University of Jerusalem

**Keywords:** F-actin, *Plasmodium*, anopheles, apoptosis, caspases, live imaging, midgut invasion, mosquito, ookinete

## Abstract

The mosquito midgut is a critical barrier that *Plasmodium* parasites must overcome to complete their developmental cycle and be transmitted to a new vertebrate host. Previous confocal studies with fixed infected midguts showed that ookinetes traverse midgut epithelial cells and cause irreversible tissue damage. Here, we investigated the spatiotemporal dynamics of ookinete midgut traversal and the response of midgut cells to invasion. A novel mounting strategy was established, suitable fluorescent dye combinations were identified and protocols optimized to label mosquito tissues *in vivo*, and live imaging protocols using confocal microscopy were developed. Tracking data showed that ookinetes gliding on the midgut surface travel faster and farther than those that remain in the lumen or those that have invaded the epithelium. Image analysis confirmed that parasite invasion and cell traversal occur within a couple of minutes, while caspase activity in damaged cells, indicative of cellular apoptosis, and F-actin cytoskeletal rearrangements in cells extruded into the gut lumen persist for several hours. This temporal difference highlights the importance of hemocyte-mediated cellular immunity and the mosquito complement system to mount a coordinated and effective antiplasmodial response. This novel *in vivo* imaging protocol allowed us to continuously observe individual ookinetes in live mosquitoes within the gut lumen and during cell traversal and to capture the subsequent cellular responses to invasion in real time for several hours, without loss of tissue integrity.

**IMPORTANCE** Malaria is one of the most devastating parasitic diseases in humans and is transmitted by anopheline mosquitoes. The mosquito midgut is a critical barrier that *Plasmodium* parasites must overcome to complete their developmental cycle and be transmitted to a new host. Here, we developed a new strategy to visualize *Plasmodium* ookinetes as they traverse the mosquito midgut and to follow the response of damaged epithelial cells by imaging live mosquitoes. Understanding the spatial and temporal aspects of these interactions is critical when developing novel strategies to disrupt disease transmission.

## Abstract (precis)

Malaria is one of the most devastating parasitic diseases in humans and is transmitted by anopheline mosquitoes. The mosquito midgut is a critical barrier that *Plasmodium* parasites must overcome to complete their developmental cycle and be transmitted to a new host. Here, we developed a new strategy to visualize *Plasmodium* ookinetes as they traverse the mosquito midgut and to follow the response of damaged epithelial cells by imaging live mosquitoes. Understanding the spatial and temporal aspects of these interactions is critical when developing novel strategies to disrupt disease transmission.

## INTRODUCTION

*Plasmodium* parasites encounter several physical barriers as they develop in the mosquito vector. Motile ookinetes form within the midgut lumen and must traverse the peritrophic matrix before invading the midgut epithelium. We have previously shown that ookinete invasion peaks around 24 h postfeeding (PF) and causes irreversible damage to invaded cells (1) and triggers a series of cellular responses, such as epithelial nitration (2, 3) and caspase activation and major rearrangements of the actin cytoskeleton (1). Damaged cells are extruded into the gut lumen through constriction of an actin ring that forms on the basal side of the cell (1). Later studies showed that epithelial nitration promotes activation of the mosquito complement-like system (4) by triggering the release of hemocyte-derived microvesicles on the basal surface of invaded cells (5). A coordinated response of midgut epithelial cells, hemocytes, and the complement-like system is necessary for mosquitoes to mount an effective antiplasmodial response. Our current understanding of the cell biology of ookinete invasion is based mostly on immunofluorescent studies performed with fixed materials at preselected time points; such studies do not provide precise information on the timing of key cellular events (1). For example, it is clear that ookinetes must traverse invaded epithelial cells before the damaged cells are extruded into the midgut lumen, but the time frame of these events *in vivo* has not been well established.

The first live imaging studies of ookinete midgut invasion were done using differential interference contrast (DIC) microscopy of *in vitro*-cultured Plasmodium gallinaceum (bird malaria) ookinetes placed on top of fragments of dissected Aedes aegypti midguts (6). This method involves extensive manipulation of the mosquito midgut that can result in tissue injury. The samples were imaged for a maximum of 3 h due to the limited viability of dissected midguts. Many ookinetes were observed gliding on the luminal surface of epithelial cells, and the images documented that ookinetes are flexible and can suffer temporary constriction as they move through the microvillus-associated network. DIC imaging allowed rapid temporal resolution at 5-to-15-s intervals, and the images documented that it takes an average of 1.3 min from the onset of cell penetration for ookinetes to complete internalization. They also showed that ookinete-invaded cells underwent caspase activation and apoptosis (6). However, spatial resolution in the “Z” axis was limited because ookinetes could not be visualized once they had invaded the cell.

A second imaging study dissected Anopheles gambiae and Anopheles stephensi midguts infected with green fluorescent protein (GFP)-labeled Plasmodium berghei parasites. Midguts could be imaged for a maximum of only 30 min using confocal microscopy due to loss of viability following midgut dissection (7). The gut epithelium was fluorescently labeled by incubation of tissues *ex vivo* with a lipophilic dye that stained only the basal membrane of epithelial cells. Examination of the results confirmed that ookinetes predominantly use an intracellular route to traverse the midgut epithelium and that ookinetes can serially traverse the cytoplasm of several midgut cells before egressing to the basolateral intercellular space to reach the basal lamina. In addition, ookinetes were observed gliding on the membrane foldings of the basolateral labyrinth, and wound repair following epithelial extrusion into the gut lumen involved extensive lamellipodia crawling from adjacent cells. The ookinetes were subjected to three-dimensional (3D) visualization and exhibited different modes of motility, including stationary rotation, translocational spiraling, and straight‐segment motility (7). However, because the midgut luminal membrane was not labeled, it was not possible to capture the initial invasion events.

In this study, we labeled the midgut of Anopheles gambiae G3 females with specific fluorescent markers *in vivo* to capture the full invasion process. New imaging methods were developed to minimalize the processing of mosquitoes, as well as to increase the viability of tissues, allowing longer time frames of live-tissue imaging. This facilitated the capture of both ookinete midgut traversal and epithelial cell responses to parasite invasion.

## RESULTS

### Sample preparation for live *in vivo* imaging.

**FIG 1:**
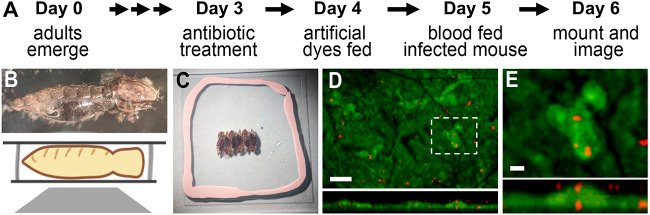
Sample preparation for live *in vivo* imaging of mosquitoes. (A) Experimental design for introduction of fluorescent markers inside mosquitoes. (B) (Top panel) Head, wings, and legs of mosquitoes are removed prior to mounting. (Bottom panel) Side view schematic of a mosquito mounted and imaged on an inverted microscope. (C) Six engorged mosquitoes were aligned and gently pressed between a cover slip and glass slide using adhesive putty. (D) XY image (top) and XZ image (bottom) of the mosquito midgut (green) infected with P. berghei-mCherry (red). Scale bar = 50 μm. (E) Magnified image from box in XY image in panel D showing cells (green) invaded by *Plasmodium* (red). Bottom panel, XZ image. Scale bar = 10 μm.

Two major obstacles had to be overcome to image *Plasmodium* ookinete midgut invasion in real time. The first obstacle entailed developing a new strategy to mount blood-fed infected mosquito midguts while maintaining tissue viability for several hours. Our initial attempts to mount dissected midguts *ex vivo* were unsuccessful due to the loss of tissue integrity. Dissected midguts could not be imaged for more than 20 to 30 min, in agreement with previous reports (7). Levels of ookinete invasion peak at 24-h post-blood meal, a time when the mosquito midgut is under physical stress because of mechanical stretching to accommodate the blood meal and when it can also be damaged by the extensive secretion of digestive proteases. To solve this problem, whole-blood-fed mosquitoes, infected by feeding on mice infected with P. berghei-mCherry parasites (Fig. 1A), were mounted after decapitation and removal of legs and wings to immobilize them (Fig. 1B, top panel). Previous studies have shown that decapitated mosquitoes can survive for long periods of time, even for several days (8). Imaging whole mosquitoes allowed us to maintain an adequate oxygen supply to the gut through the tracheal respiratory system. To minimize any possible injury, mosquitoes were gently knocked down using CO_2_ (instead of using air suction followed by ice cooling) and were placed sideways to enable imaging of the midgut through the thinnest and lightest areas of the abdominal cuticle. Mosquitoes were immobilized using malleable adhesive craft putty as a spacer, to confine them to the narrow space between the glass slide and a coverslip (Fig. 1C). Using this mounting setup, we were able to image the blood-fed midgut through the cuticle for more than 4 h using fluorescent confocal microscopy (Fig. 1D and E).

The second obstacle was the difficulty of identifying appropriate fluorescent markers to label midgut epithelial cells in P. berghei-infected mosquitoes. These markers must retain strong fluorescence for more than 24 h (time of peak ookinete invasion after blood feeding) at low concentrations, to avoid toxicity. Blood-fed mosquitoes refrain from subsequent feeding until digestion is complete; therefore, dyes were fed in saline solution using a membrane feeder before mosquitoes were fed on P. berghei-infected mice. An alternative method for labeling mosquitoes by systemic injection was also tested for nine potentially suitable fluorescent live-cell tracer dyes (see Table 1). Membrane feeding of CFDA-SE (carboxyfluorescein diacetate succinimidyl ester) in a saline solution worked very well to label all midgut epithelial cells. This is a cell-permeative dye with low toxicity that is commonly used for long-term cell tracing (9). Epithelial caspase-3 activity was detected by feeding NucView to mosquitoes, a substrate that, when cleaved by caspase-3, releases a high-affinity DNA binding dye which produces nuclear fluorescent staining (10). Epithelial F-actin was visualized *in vivo* by feeding mosquitoes SiR-actin, and this made it possible to follow the reorganization of epithelial actin after ookinete invasion. Systemic injection of the lipophilic membrane dye DiD labeled hemocytes very efficiently, as previously reported (5). These dyes were often used in combination by feeding them together. Labeling multiple components in the same mosquito made it possible to obtain a more comprehensive picture of mosquito-parasite dynamics *in vivo*.

**TABLE 1:** Fluorescent dyes tested *in vivo* for this study

Name^*a*^	Marker	Administration	Notes
Calcein	Live cells	Feeding, injection	Fades due to diffusion from cells
Cell Mask	Membrane	Feeding, injection	Fades due to internalization
*CFDA-SE	Live cells	Feeding	Good labeling
DAF-FM diacetate	Nitric oxide	Feeding	Weak and inconsistent signal
*DiI family	Hemocytes	Injection	Good labeling but poor mosquito recovery
Ethidium homodomer-2	Dead cells	Feeding	High background signal
*NucView	Caspase-3 activity	Feeding	Good labeling
*SiR-actin	F-actin	Feeding	Good labeling
SYTO	Nuclei	Feeding, injection	High background signal

a Dyes that gave good labeling were used in further experiments and are marked with an asterisk (*). DAF-FM diacetate, diaminofluorescein-FM diacetate.

### Ookinetes have different fates after they develop in the midgut lumen.

Knowing that P. berghei ookinete midgut invasion peaks around 24 h postfeeding (PF), we imaged infected midguts from 22 to 26 h PF to maximize the probability of capturing ookinete dynamics before, during, and after midgut invasion. Z-stack sections were obtained at 1-μm intervals, every 30 s, to capture quick ookinete movements and obtain cross sections that encompassed the full thickness of the mosquito epithelium, from the midgut lumen up to the basal side of epithelial cells. Images were imported into Imaris, a visualization software program, to perform tracking analysis of the ookinetes (see Fig. S1A in the supplemental material).

10.1128/mSphere.00692-20.1 FIG S1 (A) Tracking analysis (yellow lines) of ookinetes (white spheres) from a single midgut (green). Some parasites remained as immobile zygotes (red) and were not tracked. (B) NucView488 caspase signal (green) in midguts infected (left) with parasites (red) or left uninfected (right). Both snapshots were taken at 26-h post-blood meal. Scale bar = 20 μm. Download FIG S1, DOCX file, 8.7 MB. Copyright © 2020 Trisnadi and Barillas-Mury. 2020 Trisnadi and Barillas-Mury This content is distributed under the terms of the Creative Commons Attribution 4.0 International license.

**FIG 2:**
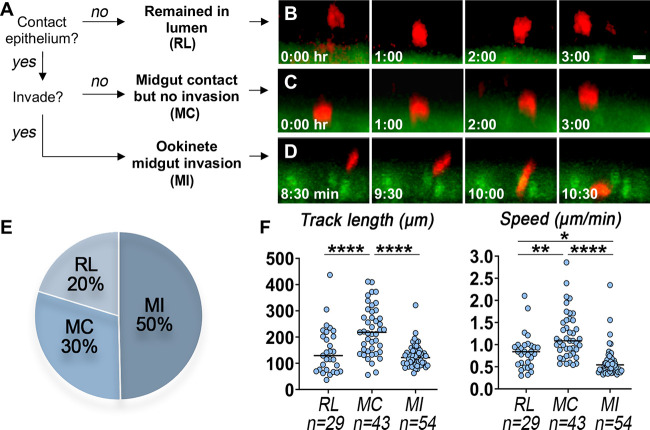
Tracking analysis of ookinetes during live *in vivo* mosquito imaging. (A to D) Tracked ookinetes (A) were categorized into three possible fates: (B) remaining in lumen (RL), (C) midgut contact without invasion (MC), and (D) midgut invasion (MI). Scale bar = 5 μm. (E) Percentages of ookinetes with outcomes corresponding to each of the fates. (F) Total distance traveled (left) and speed (right) for RL, MC, and MI ookinetes. *, *P* < 0.05; **, *P* < 0.01; ****, *P* < 0.0001.

We tracked 143 individual ookinetes from four mounted midguts and found that the parasites had three possible fates. Although 50% (71/143) of them (see Movie S1 in the supplemental material) invaded the midgut, others (30%; 43/143) came in contact with the midgut surface (Movie S2) but did not invade the cell and 20% (29/143) were motile but remained within the gut lumen (Movie S3) and never made contact with the midgut surface (Fig. 2A to E). Any spherical parasite that lacked the characteristic “banana shape” of ookinetes was presumed to be a zygote and was not included in this analysis. A successful cell invasion event does not guarantee that the parasite will reach the basal lamina and develop into an oocyst. Of the 71 ookinetes that invaded the midgut, 76% (54/71) had already reached the basal lamina and remained there for the duration of the imaging (4 h), 11% (8/71) egressed back into the lumen of the gut and we were able to track 9 ookinetes (13%) as they actively traversed the epithelial cells. We also estimated the time required for an ookinete to traverse the midgut. Because we scanned the tissues every 30 s, we were able to determine that it took these 9 parasites between 60 and 120 s to traverse epithelial cells, with a median of 90 s and an average of 83 s (see Table S1A in the supplemental material).

10.1128/mSphere.00692-20.2 TABLE S1 (A) Time of ookinete traversal of mosquito midgut epithelial cells. (B) Activation of caspase activity in mosquito midgut epithelial cells in response to ookinete invasion. (C) Duration of actin reorganization in mosquito midgut epithelial cells in response to ookinete invasion. (D) Duration of increase in F-actin levels in midgut epithelial cells in response to ookinete invasion. (E) Duration of decrease in F-actin levels in midgut epithelial cells in response to ookinete invasion. Download Table S1, XLSX file, 0.1 MB. Copyright © 2020 Trisnadi and Barillas-Mury. 2020 Trisnadi and Barillas-Mury This content is distributed under the terms of the Creative Commons Attribution 4.0 International license.

10.1128/mSphere.00692-20.3 MOVIE S1 Ookinete midgut invasion. Ookinete (red) invasion into the midgut (green) in XY view (top) and XZ view (bottom) is shown. Download Movie S1, MOV file, 2.9 MB. Copyright © 2020 Trisnadi and Barillas-Mury. 2020 Trisnadi and Barillas-Mury This content is distributed under the terms of the Creative Commons Attribution 4.0 International license.

10.1128/mSphere.00692-20.4 MOVIE S2 Ookinete that contacted midgut. An ookinete (red) that contacted the midgut (green) but did not invade within the imaging timeframe is shown (XY view on top and XZ view on bottom). Download Movie S2, MP4 file, 6.2 MB. Copyright © 2020 Trisnadi and Barillas-Mury. 2020 Trisnadi and Barillas-Mury This content is distributed under the terms of the Creative Commons Attribution 4.0 International license.

10.1128/mSphere.00692-20.5 MOVIE S3 Ookinete that remained in lumen. An ookinete (red) that never contacted the midgut (green) during the imaging timeframe is shown (XY view on top and XZ view on bottom). Download Movie S3, MP4 file, 3.7 MB. Copyright © 2020 Trisnadi and Barillas-Mury. 2020 Trisnadi and Barillas-Mury This content is distributed under the terms of the Creative Commons Attribution 4.0 International license.

We then compared the motility of the 54 ookinetes that reached the basal lamina with that of parasites that remained in the gut lumen (*n* = 29) or that only came in contact with the microvillar surface but did not invade the cell (*n* = 43). A circular spinning motion was often observed in ookinetes gliding on the gut surface (Movie S1), and those parasites displaced significantly longer distances of traversal (ANOVA, *P* < 0.0001) (Fig. 2F, left) and moved faster (ANOVA, *P* < 0.0001) (Fig. 2F, right) than the ookinetes after they invaded the midgut or than those that moved within the blood bolus but did not contact the gut surface.

### Caspase activation in invaded midgut cells.

We also investigated the kinetics of midgut epithelial cell apoptosis in response to ookinete invasion by tracking caspase activity in cells invaded by ookinetes. NucView was fed to mosquitoes in a saline solution prior to *Plasmodium* infection. Caspase signal was not detected in midguts of control mosquitoes fed on a healthy mouse, indicating that apoptosis of midgut cells is rare under these conditions, while it was readily observed following ookinete invasion (Fig. S1B).

**FIG 3:**
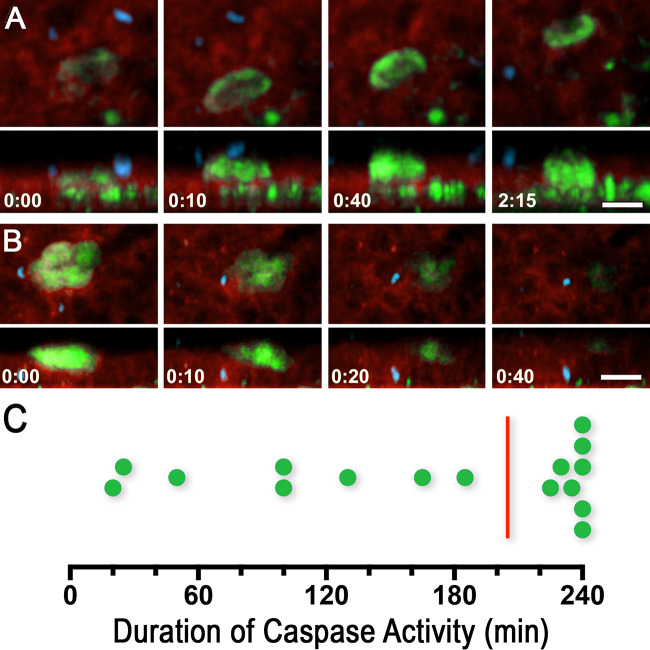
Caspase activity is dynamic in infected midguts. (A) Snapshots of a cell increasing in caspase activity (green) (midgut in red, ookinetes in blue). Time stamps in hours and minutes from the moment the signal could first be detect until it reached maximum signal. Scale bar = 20 μm. XY view on top panels and XZ view on bottom. (B) Snapshots of cells decreasing in caspase activity (green). Timestamps in panels A and B represent the amount of time (hours:minutes) that elapsed from the moment that the cell reached maximum signal until it was no longer detectable. Scale bar = 20 μm. (C) Distribution of the durations of caspase signal in minutes. Several cells were positive for caspase for most of the 4-h imaging session. The vertical red line indicates the median.

Caspase activity was detected in a total of 16 single cells or cell clusters from four P. berghei-infected midguts that were imaged for 4 h. Interestingly, the NucView signal was cytoplasmic and not nuclear, indicative of cell damage and DNA release into the cell cytoplasm of invaded cells. A relative level of signal intensity was established for each midgut, where the strongest signal was 100% and the weakest 0%. A minimum threshold was applied, according to which caspase activity with signal intensity that was at least 20% of the highest in the sample was considered to represent a positive result (Fig. 3A and B; see also Table S1B) (Movies S4 and S5). Most of the 16 cells (13/16 = 81%) were already positive for caspase activity when the imaging started (Fig. 3B; see also Table S1B), and 8 cells were still positive when the imaging ended after 240 min (Fig. 3A; see also Table S1B). In 8 cells, caspase activity was detected for close to 4 h (225 to 240 min) (Fig. 3C; see also Table S1B).

10.1128/mSphere.00692-20.6 MOVIE S4 Caspase activity increase. A cell increasing in caspase activity (green) is shown (midgut in cyan, ookinetes in red). Download Movie S4, MOV file, 0.3 MB. Copyright © 2020 Trisnadi and Barillas-Mury. 2020 Trisnadi and Barillas-Mury This content is distributed under the terms of the Creative Commons Attribution 4.0 International license.

10.1128/mSphere.00692-20.7 MOVIE S5 Caspase activity decrease. A cell decreasing in caspase activity (green) is shown (midgut in cyan, ookinetes in red). Download Movie S5, MOV file, 0.4 MB. Copyright © 2020 Trisnadi and Barillas-Mury. 2020 Trisnadi and Barillas-Mury This content is distributed under the terms of the Creative Commons Attribution 4.0 International license.

### Actin reorganization in response to ookinete invasion.

Previous confocal studies with fixed tissues showed that the actin cytoskeleton undergoes dramatic rearrangements as cells damaged by *Plasmodium* invasion are extruded from the midgut epithelial layer, by forming an actin ring at the base of damaged cells that is gradually constricted (1). Because traditional actin markers, such as phalloidin, are highly toxic (11) and impermeable to live cells, we decided to use the F-actin-specific SiR-actin fluorophore (12).

**FIG 4:**
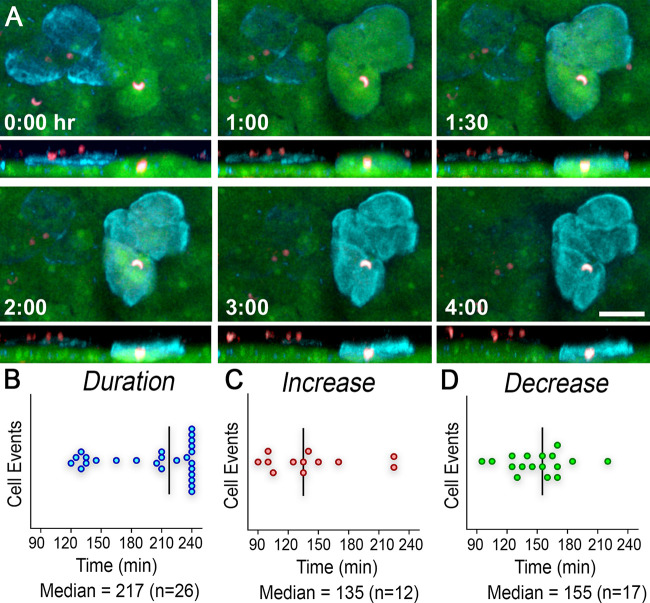
Actin undergoes rearrangement in invaded midgut cells. (A) Snapshots of two cell clusters invaded by ookinetes (red) and undergoing actin rearrangement (cyan) as seen in XY (top) and XZ (bottom) views. The cell cluster on the left had decreasing F-actin levels, while the cells on the right had increasing F-actin levels (midgut in green). Timestamps represent passage of time in hours after imaging start. Scale bar = 30 μm. (B) Duration of F-actin signal in single cells or cell clusters with sustained activity. (C) Time it took for F-actin to reach maximum levels. (D) Time it took for F-actin to decrease back to basal levels. The horizontal black lines indicate the median.

Midgut cells invaded by *Plasmodium* ookinetes exhibited strong F-actin staining. These damaged cells could be seen protruding into the lumen of the midgut as they were extruded from the epithelial sheet (Fig. 4A; see also Movie S6). Five midguts were imaged for 4 h starting at 24 h PF. A total of 34 single cells or cell clusters with F-actin signal were observed, and a 20% minimal signal threshold was also applied. The median duration of positive F-actin signal was 217 min for 26 cells that were imaged for a total of 240 min (Fig. 4B; see also Table S1C). We also established how long it took for a cell to achieve maximum F-actin levels and for it to decrease to background levels. We captured the appearance of F-actin in 12 invasion events, and F-actin levels increased over a median period of 135 min (Fig. 4C; see also Table S1D). Once maximum levels were reached, it took about 20 min longer for F-actin to decrease to background levels, with a median of 155 min (*n* = 17) (Fig. 4D; see also Table S1E). We also found that some ookinetes that remained intracellular when F-actin levels increased were still able to egress from the cell (Movie S7A), while others are completely surrounded by F-actin and trapped inside the cell. Parasites that failed to egress were eliminated when the cell budded from the midgut epithelium (Movie S7B).

10.1128/mSphere.00692-20.8 MOVIE S6 Actin activity increase and decrease. Two cell clusters invaded by ookinetes (red) and undergoing actin rearrangement (cyan) are shown in XY (top) and XZ (bottom) views. The cell cluster on the left shows decreasing F-actin levels, while the cells on the right show increasing F-actin levels (midgut in green). Download Movie S6, MOV file, 0.4 MB. Copyright © 2020 Trisnadi and Barillas-Mury. 2020 Trisnadi and Barillas-Mury This content is distributed under the terms of the Creative Commons Attribution 4.0 International license.

10.1128/mSphere.00692-20.9 MOVIE S7 (A and B) Ookinete escaping from invaded cell and ookinete trapped inside the cell. An ookinete (red) is shown to migrate from the invaded cell, which was undergoing actin rearrangement (cyan). Midgut in green. Download Movie S7, MOV file, 34.1 MB. Copyright © 2020 Trisnadi and Barillas-Mury. 2020 Trisnadi and Barillas-Mury This content is distributed under the terms of the Creative Commons Attribution 4.0 International license.

### Hemocytes patrol the basal surface of the midgut.

We previously showed that the mosquito midgut releases prostaglandin E2 (PGE2) when epithelial cells come in contact with bacteria from the gut microbiota or with the immune elicitors that they release (13). Ookinete invasion triggers PGE2 synthesis, and this is a strong hemocyte chemoattractant (13). We investigated hemocyte dynamics following ookinete invasion in live mosquitoes. Hemocytes were fluorescently labeled by systemic injection of DiD, a lipophilic dye that specifically labels this cell population (5). The next day, mosquitoes were infected by feeding on a P. berghei-mCherry-infected mouse. We found that hemocytes were actively patrolling the basal surface of infected midguts (Movie S8A) and that a few hemocytes appeared to be tracking specific ookinetes (Movie S8B), but the specificity and functional relevance of these transient interactions remain to be determined.

10.1128/mSphere.00692-20.10 MOVIE S8 (A and B) Ookinetes and hemocyte patrolling the midgut basal surface. Labeled hemocytes in an infected midgut show high motility. Ookinetes are highlighted in red, hemocytes in cyan, and midgut in green. Some hemocytes appear to track ookinetes. Download Movie S8, MOV file, 22.7 MB. Copyright © 2020 Trisnadi and Barillas-Mury. 2020 Trisnadi and Barillas-Mury This content is distributed under the terms of the Creative Commons Attribution 4.0 International license.

## DISCUSSION

Three key components had to be integrated for successful live *in vivo* imaging of ookinetes as they traversed the mosquito midgut: development of a novel mounting protocol, identification and delivery of appropriate fluorescent markers to label mosquito cells, and establishment of a confocal microscope imaging strategy. Mounting live blood-fed mosquitoes made it possible to capture the invasion process and the midgut epithelial responses to parasite invasion by imaging each midgut continuously for several hours, without loss of tissue viability, overcoming a major limitation in previous studies. Confocal point scanning microscopy made it possible to capture the fluorescent signal from the labeled mosquito cells and the transgenic parasites, while avoiding the background signal from the mosquito cuticle, a tissue with strong autofluorescence. A 2-photon laser system was tested, but it failed because the mosquito cuticle absorbed the energy of the laser, and this damaged and sometimes even burned the sample.

Ookinetes in the lumen that contacted the midgut surface traveled the longest distances and moved faster than ookinetes that did not contact the midgut (Fig. 2F). Ookinetes were often observed spinning in circles along the luminal surface of the midgut (see Movie S1 in the supplemental material). This “spiraling” motion has been previously reported (7). Once ookinetes enter the epithelial cell, they slow down and travel a shorter distance than when they glide on the surface (Fig. 2F). This is likely due to the interaction with the actin cytoskeleton of the epithelial cell, a barrier that the parasite must overcome to traverse the midgut. A preliminary study using live *in vivo* imaging of dissected midguts also showed similar kinetics of P. berghei ookinetes in A. gambiae and A. stephensi, with slower motility as parasites crossed the epithelium and faster displacement when they glided in the midgut lumen (14). We were able to observe the active process of invasion of nine parasites and found that it took them an average 83 s to traverse epithelial cells (see Table S1A in the supplemental material).

Ookinete invasion triggers a caspase-mediated apoptotic response (1), and a previous *ex vivo* study also reported higher caspase activity in invaded cells using PhiPhiLux-G_1_D_2_, a transient dye to detect caspase-3 activity (6). In the current study, NucView, a stable caspase marker, was used. The use of NucView allowed extended continuous imaging and revealed that epithelial cells damaged by the parasite induced caspase activity and were able to remain associated with the epithelium for several hours, in some cases for the 4-h duration of the imaging session (Fig. 3C; see also Table S1B).

A strong F-actin signal was also observed in ookinete-invaded cells, indicative of reorganization of the actin cytoskeleton, but was absent in uninfected midguts. In some instances, we observed cells with strong F-actin signal without a parasite in direct contact with the cell. However, ookinetes were always present in neighboring cells, suggesting that parasites can sequentially invade multiple cells, as previously reported (1) and confirmed in a recent preprint report (14). During this dynamic rearrangement of the actin cytoskeleton, a few parasites were surrounded by F-actin and trapped inside the cell (Movie S7B). Similarly to caspase activity, F-actin activity also can be detected for several hours, with a median of 217 min (Fig. 4B), and can sometimes persist for 4 h. This indicates that, under these physiological conditions (22°C), the extrusion of midgut epithelial cells takes several hours, while ookinetes move much faster, as it takes them an average of 83 s to traverse an epithelial cell. We have previously shown that caspase activation and epithelial nitration following ookinete invasion are critical for mosquitoes to activate the mosquito complement system and mount an effective antiplasmodial response (4).

Circulating hemocytes are also key players in this defense response. Ookinete invasion triggers PGE2 release by the mosquito midgut, which attracts hemocytes to the basal surface of the midgut (13). If hemocytes come in contact with a nitrated surface, they release microvesicles, promoting activation of the mosquito complement system (5). Epithelial responses are not fast enough to effectively kill ookinetes during cell traversal, but by attracting hemocytes that move to the invasion site and subsequently release microvesicles, effective complement-mediated elimination of most ookinetes can be achieved after they egress from the epithelial cell. We found instances where patrolling hemocytes appeared to track nearby ookinetes, suggesting that they may have the ability detect and target ookinetes (Movie S8A and B). However, further studies with extensive imaging would be necessary to confirm if this is indeed the case.

We developed a new protocol for live, *in vivo* imaging of *Plasmodium*-infected mosquitoes that allows direct observation of ookinetes and their interactions with the mosquito midgut as they traverse the epithelium. This method preserves the tracheal and circulatory systems, allowing oxygen and nutrient delivery to intact mosquito organs, making it possible to acquire images for several hours without loss of organ viability. The results from our time-lapse imaging and tracking analysis are in agreement with the original timebomb model, based on confocal analysis of fixed midgut samples. They provide information on the temporal dynamics of ookinete motility and midgut traversal, as well as actin rearrangements, apoptosis, and extrusion of invaded epithelial cells. This *in vivo* imaging method is versatile and can be readily adapted to study later stages of *Plasmodium* development in the mosquito, such as the oocyst and sporozoite stages, as well as other vector-pathogen combinations.

## MATERIALS AND METHODS

### Sample preparation.

A. gambiae CDC G-3 mosquitoes were reared according to standard protocols at 28°C in 80% humidity with a 12-h light/dark cycle and a 10% Karo syrup cotton ball. Three-day-old adult females were given water supplemented with penicillin (Sigma-Aldrich) (100 U/ml) and streptomycin (Sigma-Aldrich) (100 μg/ml) antibiotics overnight and were then fed the fluorescent dye of choice the next day using an artificial feeder at 38°C. Fluorescent probes were delivered in a mixture of 0.15 M NaCl and freshly prepared 0.01 M NaHCO_3_, adjusted to pH 7.3 using HCl (15). Added fluorescent dyes included CFDA-SE (Invitrogen) (1:100) to label the midgut, SiR-actin (Cytoskeleton) (1:300) for F-actin activity, and NucView488 (Biotium) (1:50) for caspase-3 activity. Only fully engorged females were kept for subsequent infection. If hemocytes were to be labeled, those mosquitoes were additionally injected with freshly diluted (1:20 in water) Vybrant DiD (Thermo Fisher). Antibiotic treatment was kept in a 10% sucrose solution after dye feeding until infection day. The following day, mosquitoes were starved in the morning and then fed in a BALB/c mouse in the afternoon before being transferred to 19°C until mounting. Mice were infected with P. berghei-mCherry at a parasitemia level of 6% to 8%.

Sample preparation started the next day at approximately 1 h before the imaging start time to allow careful mounting. To capture ookinete invasion, imaging began at 22 h PF. For experiments observing the later events of actin and caspase dynamics, imaging began at 24 h PF. Mosquitoes were placed in a CO_2_ chamber containing dry ice for knockdown and were then gently transferred to a dish on ice. Engorged females were placed in a glass well containing enough Schneider’s insect medium (S0146, Sigma-Aldrich) to keep them wet but not floating. The head, wings, and legs were removed using a blade and no. 5 forceps. For mounting, a thin strip of adhesive craft putty tape was placed along the edge of a #1.5 square coverslip. Up to 15 mosquitoes were lined up directly on the cover slip, alternating anterior and posterior ends for a compact fit and reduction in twitching. Roughly 5 μl of Schneider’s insect medium was added to coat all mosquitoes and prevent desiccation. A glass slide was then gently pressed evenly on top of the mosquitoes-putty-coverslip until the midgut could be seen to lay flat against the slide. Samples were immediately imaged, keeping them at room temperature (22°C).

### Image acquisition.

A Leica SP5 confocal microscope was used for all imaging. Experiments initially used a 20 × 0.7 numerical-aperture (NA) oil lens objective to obtain a broad overview of dynamics and to maximize the number of events captured. A few experiments were done with a 40 × 1.25 NA oil lens objective to acquire data with higher spatial resolution. A 488-nm-wavelength laser was used to excite CFDA-SE midgut and NucView488 caspase and a 633-nm-wavelength laser for SiR-actin and DiD hemocytes. For P. berghei-mCherry, 561 nm was scanned sequentially. We used a resonant scanner (8,000 Hz), which required a zoom of 1.7 for the desired configuration. Z-stacks were approximately 60 μm to encompass the epithelial layer with allowances for mosquito movements during imaging, and images were taken at intervals of 1 μm. Ookinete invasion events were imaged at 30-s intervals between z-stacks, while actin and caspase experiments were imaged every 5 min. The pinhole was 2 AU and the line average was 8.

### Image processing and analysis.

Images were analyzed using Imaris 9.0.0 from Bitplane and ImageJ. First, images were smoothed using a Gaussian filter. Next, ookinetes were detected using the Spot feature with estimated 10-μm diameter, and zygotes were removed based on morphology. From the remaining objects, ookinetes were automatically tracked using the Autoregressive Motion algorithm with maximum distance of 5 μm. Then, tracks were manually edited to add, remove, or connect the initial tracks. Only tracks that were at minimum 1 h in length were included for analysis, and drift correction was applied to correct for whole-mosquito movements. To categorize ookinetes into the three possible fates (invade, midgut contact, and lumen), tracks were initially separated by z-position and then manually verified. Finally, track statistics were exported for analysis in GraphPad Prism.

For actin and caspase experiments, changes in signal intensity were used to calculate dynamics. Average signal intensity was quantified in ImageJ and normalized for each midgut as percent signal intensity, where the highest measured signal intensity was 100% and the lowest was 0%. For analysis, a minimal threshold of 20% signal intensity was applied to consider a cell or cell cluster positive for caspase or F-actin.

### Ethics statement.

Public Health Service Animal Welfare Assurance no. A4149-01 guidelines were followed according to the National Institutes of Health Animal (NIH) Office of Animal Care and Use (OACU). These studies were done according to the NIH animal study protocol (ASP) approved by the NIH Animal Care and User Committee (ACUC), with approval ID ASP-LMVR5.
