# Supplementary material for: Live In Vivo Imaging of Plasmodium Invasion of the Mosquito Midgut
Source: mSphere. 2020 Sep 2;5(5):e00692-20. doi: 10.1128/mSphere.00692-20 (PMC7471008; doi:10.1128/mSphere.00692-20)
Supplement: FIG S1 [file mSphere.00692-20-sf001.docx]

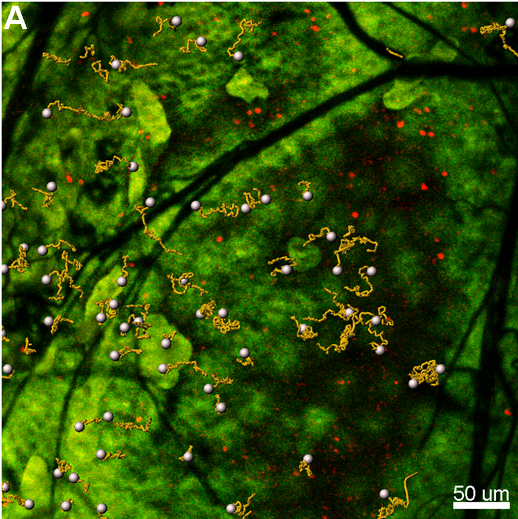


Figure S1A. Tracking analysis (yellow lines) of ookinetes (white spheres) from a single midgut (green). Some parasites remained as immobile zygotes (red) and were not tracked.


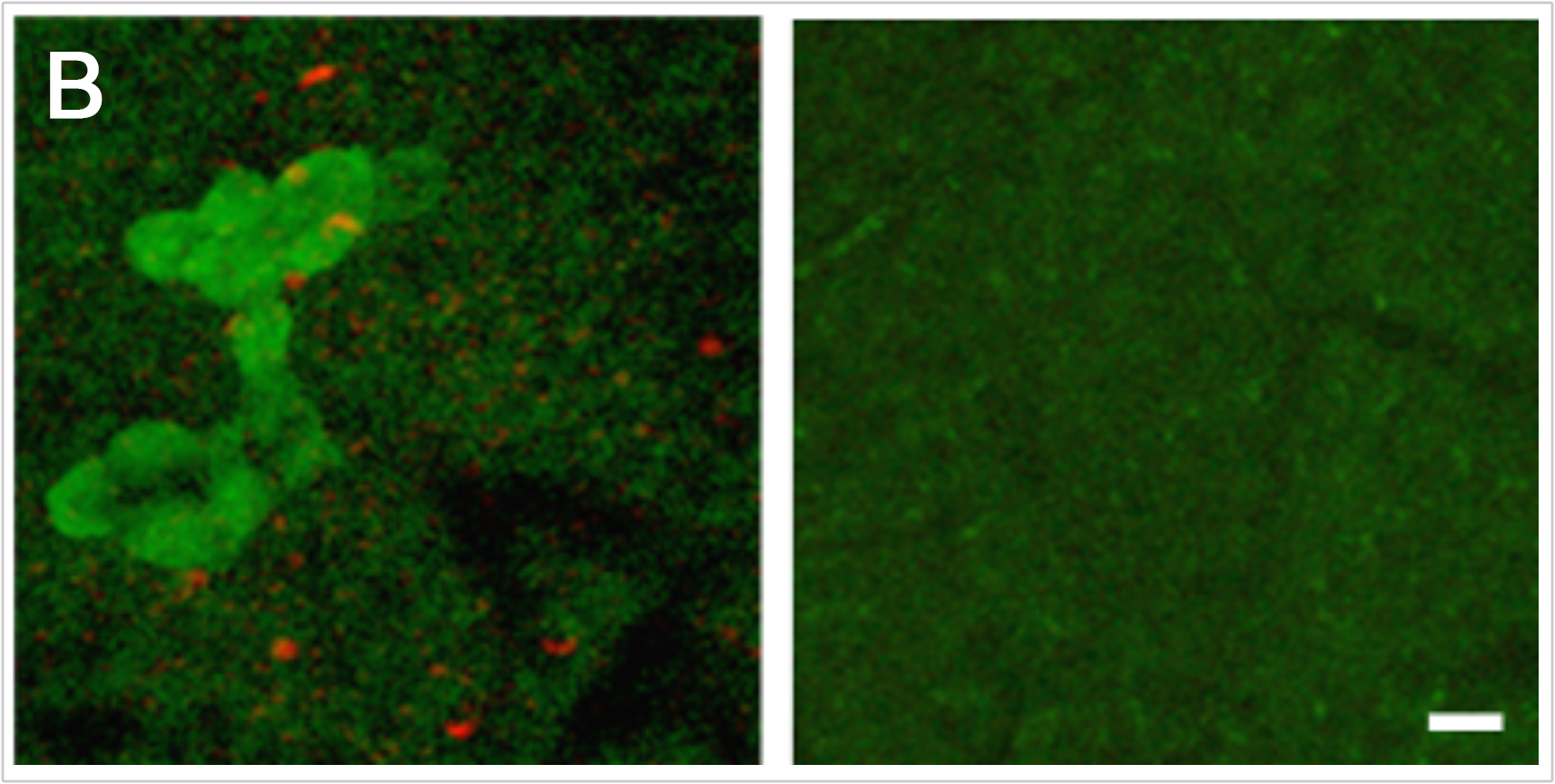


Figure S1B. NucView488 caspase signal (green) in midguts infected (left) with parasites (red) and uninfected (right). Both snapshots were taken at 26-hours post-blood meal. Scale bar = 20 µm.
